# The Spray-Dried Alginate/Gelatin Microparticles with Luliconazole as Mucoadhesive Drug Delivery System

**DOI:** 10.3390/ma16010403

**Published:** 2023-01-01

**Authors:** Marta Szekalska, Magdalena Wróblewska, Anna Czajkowska-Kośnik, Katarzyna Sosnowska, Paweł Misiak, Agnieszka Zofia Wilczewska, Katarzyna Winnicka

**Affiliations:** 1 Department of Pharmaceutical Technology, Medical University of Białystok, Mickiewicza 2c, 15-222 Białystok, Poland; 2 Department of Polymers and Organic Synthesis, Faculty of Chemistry, University of Białystok, 15-245 Białystok, Poland

**Keywords:** microparticles, sodium alginate, gelatin, luliconazole, spray drying, antifungal activity

## Abstract

*Candida* species are opportunistic fungi, which are primary causative agents of vulvovaginal candidiasis. The cure of candidiasis is difficult, lengthy, and associated with the fungi resistivity. Therefore, the research for novel active substances and unconventional drug delivery systems providing effective and safe treatment is still an active subject. Microparticles, as multicompartment dosage forms due to larger areas, provide short passage of drug diffusion, which might improve drug therapeutic efficiency. Sodium alginate is a natural polymer from a polysaccharide group, possessing swelling, mucoadhesive, and gelling properties. Gelatin A is a natural high-molecular-weight polypeptide obtained from porcine collagen. The purpose of this study was to prepare microparticles by the spray-drying of alginate/gelatin polyelectrolyte complex mixture, with a novel antifungal drug—luliconazole. In the next stage of research, the effect of gelatin presence on pharmaceutical properties of designed formulations was assessed. Interrelations among polymers were evaluated with thermal analysis and Fourier transform infrared spectroscopy. A valid aspect of this research was the in vitro antifungal activity estimation of designed microparticles using *Candida* species: *C. albicans*, *C. krusei*, and *C. parapsilosis*. It was shown that the gelatin addition affected the particles size, improved encapsulation efficiency and mucoadhesiveness, and prolonged the drug release. Moreover, gelatin addition to the formulations improved the antifungal effect against *Candida* species.

## 1. Introduction

Vulvovaginal candidiasis (VVC) is a common and multifactorial disease of the lower female reproductive tract, the primary causative agent of which is opportunistic fungi—*Candida albicans* and other associated species. *C. albicans* belongs to human microbiota and generally asymptomatically colonizes the environment of the vagina [1]. However, in the case of risk factors, such as decrease in immunity, overuse of antibiotics, pregnancy, or uncontrolled diabetes mellitus, it might lead to fungal overgrowth, epithelial invasion, and formation of virulence effector symptomatic infection and, as a consequence, exuberant mucosal inflammation [2]. The candidiasis treatment is difficult and lengthy, which is connected mainly to continuously escalating resistance of fungi; therefore, novel active substances and alternative technological solutions are still needed [3]. The vaginal mucosa is a valuable application site for local drug delivery. Nevertheless, traditional vaginal formulations possess many drawbacks, e.g., low residence time in the application site and leakage dosage form. Thus, the mucoadhesive drug delivery systems ensuring extension contact time of drug dosage forms with vaginal mucosa are a useful approach, which increase the vaginal therapy effectiveness [4]. Mucoadhesive formulations include mucoadhesive microparticles, which represent multicompartment drug delivery systems. Mucoadhesive microparticles, due to the adhesion to the mucosa at the application site and high surface area, might significantly contribute to the improvement of drug antifungal activity [5,6].

Natural polymers possess a meaningful importance in the drug delivery systems development. Sodium alginate (ALG) is a polysaccharide belonging to the natural polymers, which possesses many benefits, e.g., nontoxicity, safety, biocompatibility, and biodegradability. Its structure consists of 1,4-linked β-D-mannuronic (M), and α-L-guluronic (G) blocks linked by β (1–4) glycosidic linkages created G blocks, M blocks, and M/G units. In addition, ALG possesses good swelling and mucoadhesive properties and the ability to gel under mild conditions [7,8,9]. Although properties of ALG are significantly related to the composition and proportion of M/G blocks, ALG-based drug delivery systems might also be challenged by drawbacks, such as fragility, poor mechanical properties, and low flexibility. Thus, adequate excipients improving the properties of ALG formulations are still required. The ALG structure might be modified by the connection with cationic polymers to create polyelectrolyte complexes (PECs) [10]. There are a number of reports which prove that ALG/GEL PECs drug dosage forms are characterized by higher degradability, improved adhesion, and better mechanical and rheological properties compared with pure ALG formulations [11,12,13].

Gelatin A (GEL) is a natural high-molecular-weight polypeptide received from porcine collagen through acid hydrolysis or by degradation with using temperature or enzymes. It comprises glycine, proline, and hydroxyproline. It is categorized as Generally Recognized as Safe (GRAS) by the FDA [14]. Due to the low cost of acquisition and susceptibility of chemical structure modification, GEL is widely used in the pharmaceutical technology [11,12]. GEL from porcine skin is obtained from the acidic digestion of collagen and is referred to as type A. In the Gelatin A chain, positive charge amino groups are present, which enable interaction with the negative ALG free carboxyl group [13].

Luliconazole (LUL) is a new antifungal agent accepted by the (FDA) in 2013 for the treatment of tinea cruris, tinea pedis, and tinea corporis. The commercially available dosage form with LUL is only 1% (*w*/*w*) luliconazole cream for skin delivery. LUL is an imidazole agent, whose mechanism of antifungal activity is related to the inhibition of sterol 14α-demethylase, which disrupts ergosterol biosynthesis. LUL is included in the II class of the Biopharmaceutics Classification System (BCS), and, therefore, due to its low aqueous solubility and high permeability, elaboration delivery systems improving its therapeutic effect are needed [15,16,17].

Although the combinations of ALG and GEL have already been attempted to be used as drug carriers over the years, designing ALG/GEL microparticles by spray-drying is a novel approach. The purpose of this research was to prepare mucoadhesive and multicompartment microparticles by the spray-drying of ALG/GEL PEC mixture with LUL and evaluating the GEL addition on the quality of developed microparticles. In the next step, pharmaceutical characteristics of formulations were estimated. Moreover, swelling and mucoadhesive properties were examined. Interactions between ALG and GEL were assessed by thermal analysis and Fourier transform infrared spectroscopy. A crucial stage of this research was the in vitro antifungal activity estimation of designed microparticles using *Candida* species: *C. albicans*, *C. krusei*, and *C. parapsilosis*.

## 2. Materials and Methods

### 2.1. Materials

ALG (source of acquisition: *Macrocystis pyrifera*) with medium viscosity (282 mPa∙s for 1% solution at 25 °C, 61% mannuronic acid (M) and 39% guluronic acid (G), molecular weight 3.5 × 10^5^ Da, and M/G ratio of 1.56) was received from Sigma Aldrich. GEL from porcine skin type A was also purchased from Sigma Aldrich (St. Louis, MO, USA). LUL was provided by Kerui Biotechnology Co. LTD (Xi’an, China). Methanol and acetonitrile were attained by Merck (Darmstadt, Germany). Water was obtained by distillation using Milli-Q Reagent Water System (Billerica, MA, USA). Simulated vaginal fluid (SVF, pH = 4.2) was obtained by dissolving in 1 L of water: 5 g glucose, 2 g lactic acid, 3.51 g natrium chloride, 1.40 g potassium hydroxide, 1.0 g acetic acid, 0.4 g urea, 0.222 g calcium hydroxide, 0.16 glycerol, and 0.018 g bovine albumin [18]. Sabouraud dextrose agar (SDA) and stock cultures of *Candida albicans* ATCC^®^ 10231, *Candida krusei* ATCC^®^ 6528, and *Candida parapsilosis* ATCC^®^ 22019 from American Type Culture Collection were provided from Biomaxima (Lublin, Poland). Nylon membrane filters (0.45 µm) were received from Alchem (Toruń, Poland). Cellulose acetate membrane filters (0.45 µm) were purchased by Millipore (Billerica, MA, USA). Porcine vaginal mucosa was received from a veterinary service (Turośń Kościelna, Poland). Fragments of porcine vaginal mucosa were frozen at −20 °C and were stored up to a maximum of one month prior to testing. This process did not require the confirmation of the Local Ethical Committee for Experiments on Animals. All other reagents applied in the experiments were of analytical grade.

### 2.2. ALG/GEL Complex

In the first step, blends of polymers with constant concentration of ALG and various concentrations of GEL were created. The concentrations of the component polymers were chosen taking into account the viscosity of prepared solutions as the spray-drying of the solutions with high viscosity is limited. A 2% ALG solution was obtained by dissolving ALG in distilled water at room temperature using RZR 2020 mechanical stirrer (Heidolph Instruments, Schabach, Germany). In addition, 0.25%, 0.5%, and 0.75% GEL solutions were procured by adding different amounts of GEL to the distilled water, and the dissolution was improved by heating the solution at 60 °C for 1 h and stirring by using mechanical stirrer. When homogenous solution was received, it was cooled down to 40 °C [19]. A mixture of ALG–GEL complexes was obtained by mixing prepared constituent solutions at 40 °C.

### 2.3. Viscosity Measurement

Measurements were performed using rotational viscometer Haake Viscotester 6 Plus (Thermo Fisher Scientific, Waltham, MA, USA) at room temperature (22 ± 2 °C) with speed in the range of 5–200 rpm. Tests were conducted for 1 min, and then viscosity values were observed.

### 2.4. Turbidity Measurement

Turbidity measurement was carried out by using a Hach Model 2100 N IS^®^ Laboratory Turbidimeter (Hach Company, Loveland, CO, USA) and expressed in a nephelometric turbidity unit (NTU). The 30 g of 1% ALG, 0.125%, 0.250%, 0.375% GEL, and mixtures of 1% ALG/0.125% GEL, 1% ALG/0.250% GEL, and 1% ALG/0.375% GEL solutions were transferred to the cells, and turbidity was examined.

### 2.5. Microparticles Preparation

Microparticles were obtained by the spray-drying of ALG/GEL solutions utilizing Mini Spray Dryer B-290 (Büchi, Switzerland) (Table 1). To formulate LUL-loaded microparticles, active substance was uniformly dispersed in ALG/GEL solutions and then spray-dried. After optimization, the spray-drying parameters of the process included: temperatures inlet 135 °C and outlet 70 °C, pressure 60 mm Hg, aspirator blower capacity 100%, and feed rate 3.6 mL/min.

### 2.6. Evaluation of Microparticles Morphology

Scanning electron microscope (SEM) with high vacuum mode and secondary electron detector (Inspect™S50, FEI Company, Hillsboro, OR, USA) was applied to evaluate morphology and shape of designed ALG/GEL microparticles. Before analysis, microparticles were covered with a 6 nm gold layer. SEM analysis was conducted under 10 kV voltage and with 10 mm detector working distance. The microparticles were observed under magnifications of 2000×, 5000×, 10,000×, and 20,000×. All microparticles formulations were also examined by an optical microscope (Motic BA 400, Moticon, Wetzlar, Germany) and observed under 40× magnification. The microparticles size distribution and polydispersity index were examined by Zetasizer NanoZS90 (Malvern Instruments, Malvern, UK) after suspending in ethanol 99.8% (because in this medium, microparticles were stable).

### 2.7. Estimation of LUL Loading, Encapsulation Efficiency, and Production Yield

To evaluate loading of LUL, 10 mg of microparticles was dissolved in mixture of distilled water and ethanol in the ratio 1:9 and agitated for 1 h at 75 rpm in a water bath (37 ± 1 °C). After filtration, solution was diluted with phase (acetonitrile:water 60:40, *v*/*v*) and evaluated by the HPLC technique featured in the point 2.7.1.

Drug loading (L) was computed using the formula:

L = Q_m_/W_m_ × 100 (1)

where Q_m_ is drug encapsulated in the microparticles, and W_m_ is microparticle weight.

The mean drug encapsulation efficiency (EE) was calculated by the expression:

EE = Q_a_/Q_t_ × 100 (2)

where Q_a_ is the actual drug content, and Q_t_ is the theoretical drug content.

Yield of production (Y) was determined by the formula:

Y = W_m_/W_t_ × 100 (3)

where W_m_ is weight of microparticles, and W_t_ is theoretical weight of drug and polymer [20].

#### LUL High-Performance Liquid Chromatography (HPLC) Analysis

LUL content in the microparticles was evaluated by the HPLC method using an Agilent Technologies 1200 system (Agilent, Waldbronn, Germany), which was equipped with a column Poroshell^®^ 120 EC-C18 2.7 μM ODS 4.6 × 150 mm, 2.7 μm. Mixture of acetonitrile:water in the ratio 60:40, (*v*/*v*) as the mobile phase was applied and 0.5 mL/min of flow rate was used [21]. Analysis was performed at wavelength of 300 nm. The LUL peak was noted at 4.8 min. The standard calibration curve was linear and characterized by the correlation coefficient (R^2^) 0.999.

### 2.8. Swelling Capacity

The swelling capacity was determined using SVF (pH = 4.2). The amount of 20 mg of microparticles were situated in the baskets from USP dissolution equipment and placed in the twenty-five millilitre beakers with the 15 mL SVF. Tests were performed at the temperature 37 ± 1 °C. After time sections, the baskets were taken out of the beakers and carefully drained. In the next step, baskets with microparticles were weighted using the analytical balance, and the swelling ratio (SR) was computed by applying the expression [22]:

SR = (Ws − W_0_)/W_0_ (4)

where W_0_ is the microparticles initial weight, and W_S_ is the weight of swollen microparticles. The study was performed in triplicate.

### 2.9. Mucoadhesive Properties

Estimation of the mucoadhesive ability was conducted using TA.XT. Plus Texture Analyser (Stable Micro Systems, Godalming, UK). As the mucoadhesive layer, porcine vaginal mucosa was applied. After moisturizing with 50 µL of SVF, 100 mg of microparticles was exposed to contact with mucoadhesive layer with 1 N force for 60 s. Process parameters were as follows: pre-test speed, test speed, and post-test speed of 0.5, 0.1, and 0.1 mm/s, respectively. Experiments were conducted at 37 ± 1 °C. Mucoadhesive properties were expressed as the detachment force (F_max_, recorded by Texture Exponent 32 software, version 5.0), and the work of mucoadhesion (W_ad_) was determined from the area under the force graph vs. distance curve.

### 2.10. In Vitro LUL Release Estimation

To estimation LUL in vitro release dissolution, basket apparatus (Erweka Dissolution Tester Type DT 600 HH, Heusenstamm, Germany) was applied. Microparticles were placed in beakers containing 500 mL of SVF (pH 4.2) and stirred at 75 rpm at 37 ± 1 °C for 24 h. To receive *sink* conditions, 1% SDS was added. Samples were taken at the time intervals: 0.5, 1, 2, 3, 4, and 6 h, and analysed spectrophotometrically by using Genesys 10S UV-Vis (Thermo Scientific, Waltham, MA, USA) at 300 nm.

### 2.11. Mathematical Modeling of LUL Release Profile

To evaluate the LUL release mechanism, results received from the LUL release experiment were estimated by using various mathematical models [23]:

Zero-order kinetic:

F = k × t(5)

First-order kinetic:

lnF = k × t(6)

Higuchi model:

F = kt^1/2^ (7)

Korsmeyer–Peppas model:

F = kt^n^ (8)

Hixson–Crowell model:

1 − (1 − F)^1/3^ = kt (9)

where F is released drug, k is the constant related with the drug release, and t is the time.

### 2.12. Thermal Analysis

Thermogravimetric analyses (TGA) and differential scanning calorimetry analyses (DSC) of the unprocessed ALG, GEL, LUL, and spray-dried microparticles were conducted by using a Mettler Toledo Star TGA/DSC unit. In TGA analysis, 3–5 mg samples were placed in aluminum oxide crucibles and heated from 50 °C to 500 °C at 10 °C/min under an argon; an empty pan was used as the reference. In turn, in DSC analysis, aluminum crucibles with 3–5 mg weighted samples were heated from 0 °C to 450 °C at 10 °C/min under an argon; an empty pan was used as the reference.

### 2.13. Attenuated Total Reflectance–Fourier Transform Infrared Spectroscopy (ATR–FTIR)

All spectra were obtained using a Thermo Scientific Nicolet 6700 FTIR spectrophotometer (Waltham, MA, USA) equipped with diamond Attenuated Total Reflectance. The spectra were compared with the background spectra, and 32 scans in the range between 500 cm^−1^ and 4000 cm^−1^ were performed.

### 2.14. Antifungal Activity

To estimate the antifungal activity of the prepared microparticles, the plate diffusion technique was applied. Petri dishes with Sabouraud dextrose agar (SDA) were seeded with 50 µL of the fungal inoculums prepared using sterile 0.9% NaCl solution, with the final density 5 × 10^4^ CFU/mL (corresponding to 0.5 on the McFarland scale) [24]. After drying, 5 mm diameter wells were cut out in agar plates, where 10 mg of microparticles of all tested formulations were situated and immersed with 20 µL SVF. In addition, 50 µL of solution obtained by dissolving LUL in DMSO (corresponding to 4 mg of LUL), and DMSO were used as controls. Petri dishes with samples were incubated at 37 ± 0.1 °C for 24 and 48 h, and then the growth inhibition zones were determined by applying a caliper (Mitutoyo, Kawasaki, Japan) with an accuracy of 0.1 mm.

### 2.15. Statistics

Data were assessed by Statistica 10.0 (StatSoft, Tulsa, OK, USA) using one-way analysis of variance (ANOVA) or a Kruskal–Wallis test. Obtained results were introduced as the mean and standard deviation. The three-dimensional (3D) response surface was acquired from Statistica 10.0 to obtain the points of optimum production yield and optimum particle size.

## 3. Results and Discussion

Vulvovaginal candidiasis (VVC), despite the continuous and significant development of medicine, still constitutes an important therapeutic issue. Insufficient therapeutic effects are related mainly to the rapidly increasing resistance generated by fungal cells and limited number of effective antifungal substances. The application of multicompartment mucoadhesive drug dosage forms due to the prolonged contact of active substance with the vaginal mucosa might be a promising strategy to improve VVC treatment [1,2,3].

**Figure 1:**
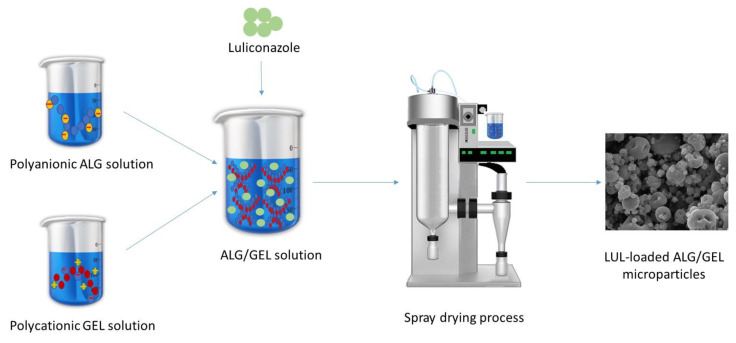
Scheme for LUL-loaded ALG/GEL microparticles preparation.

ALG negative carboxylic groups might interact with positive groups present in the GEL molecule [19]. In the first step, an interaction between the polymers occurs, which was confirmed by the increase in viscosity of ALG/GEL. Viscosity of solutions applied in the spray-drying technique is limited and, therefore, for the preliminary studies, three GEL concentrations—0.125, 0.250, and 0.375—were selected. Figure 1 presents a scheme for the LUL-loaded ALG/GEL microparticles preparation.

### 3.1. Viscosity and Turbidity

**Figure 2:**
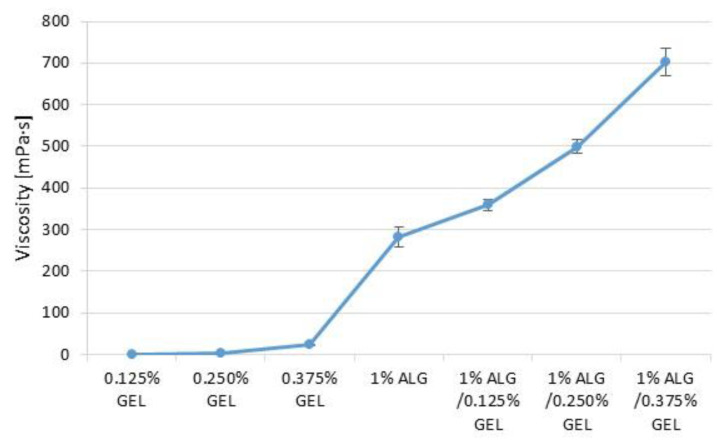
Viscosity values of 0.125%, 0.250%, 0.375% GEL, 1% ALG, and ALG/GEL PECs mixtures with regard to various polyanion/polycation ratios.

**Figure 3:**
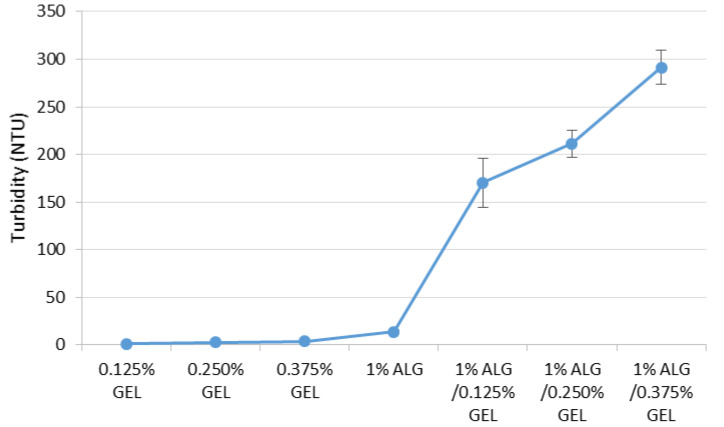
Turbidity values of 0.125%, 0.250%, 0.375% GEL, 1% ALG, and ALG/GEL PECs mixtures with regard to various polyanion/polycation ratios.

The objective of the study was to indicate formation of polyelectrolyte complexes (PECs) between ALG anionic groups and GEL cationic groups (Figure 2). Viscosity values of prepared ALG, GEL, and ALG/GEL PECs solutions are presented in Figure 2. It was observed that ALG/GEL solutions were characterized by higher viscosity that constituent polymers solutions. In addition, viscosity values increased when the GEL concentration was increased. The obtained data from the turbidity measurements showed that ALG/GEL complexes were characterized by higher values of turbidity than turbidity of solutions of constituent polymers (Figure 3). This fact confirms ALG/GEL PECs formation as a result of electrostatic interaction between negatively charged carboxylic acid groups of ALG and amino groups of GEL with positive charge [25]. Moreover, ALG/GEL complexes’ turbidity was increased with increasing GEL concentration—greater concentration of GEL led to creation of larger amount of PEC.

### 3.2. Microparticles Characteristics

**Figure 4:**
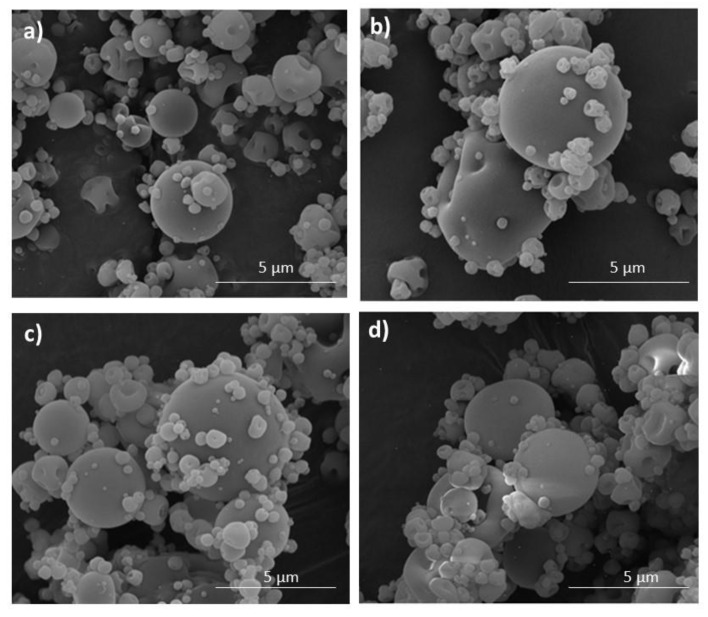
Representative images of microparticles formulation F1 (**a**), F4 (**b**), FL1 (**c**), and FL4 (**d**) under magnification 20,000×.

Microparticles morphology examined by SEM analysis is presented in Figure 4. The obtained results expressed that ALG/GEL solutions might be successfully used to develop microparticles by a one-step spray-drying technique. It was shown that both ALG microparticles placebo (F1) and LUL-loaded ALG microparticles (FL1) possessed uniform and smooth surfaces with characteristic minor protuberances (Figure 4a). The surfaces of ALG/GEL microspheres were characterized by more irregular shapes compared with pure ALG microparticles. This fact might be related with the rapid loss of water from ALG and GEL in the spray-drying process [26]. LUL-loaded microparticles possessed a more spherical shape with an insignificant number of pores than the placebo formulations (Figure 4c,d).

**Figure 5:**
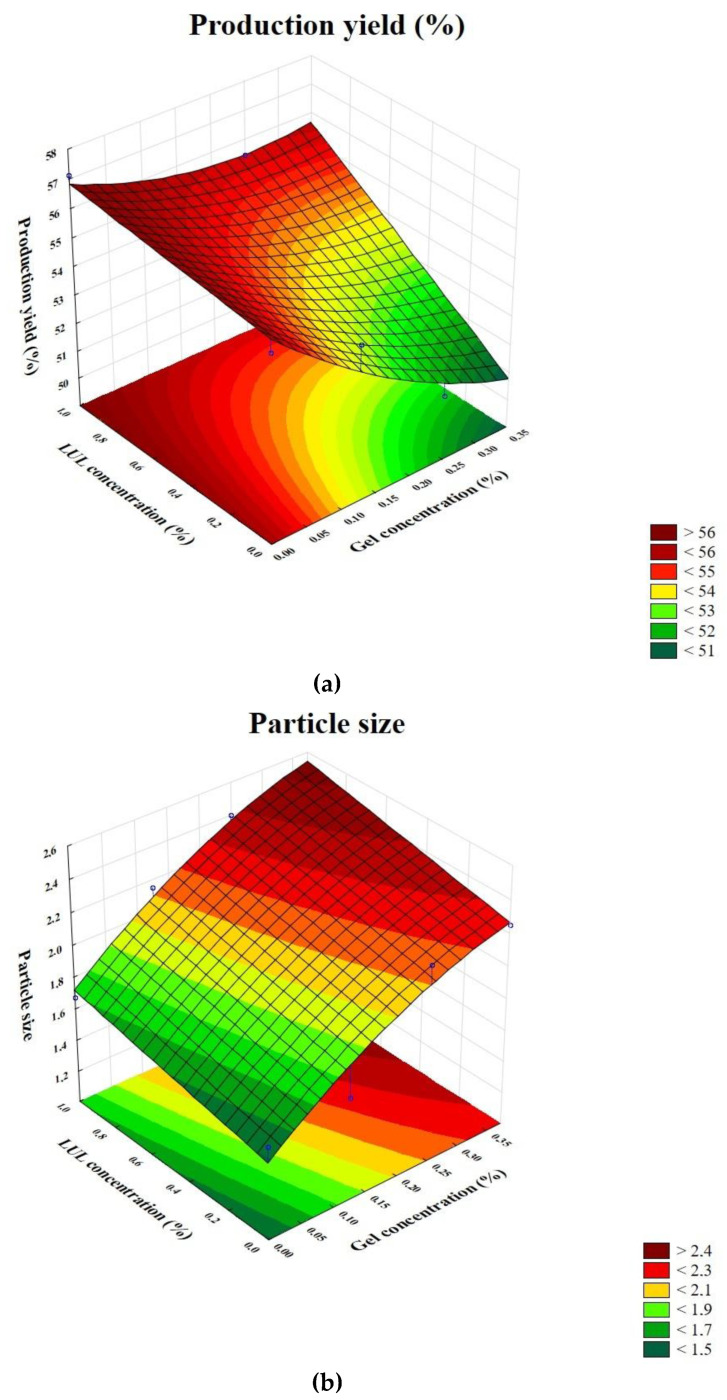
Three-dimensional response surface plots: (**a**) production yield vs. LUL presence and GEL concentrations and (**b**) particle size vs. LUL presence and GEL concentrations.

The quality evaluation of obtained ALG and ALG/GEL microparticles was performed. The tests included examination of production yield, particles mean diameter, moisture content, LUL percent loading, and LUL encapsulation efficiency (Table 2). It was shown that the spray-drying technique enabled the reception of microparticles, with production yield in the range from 50.60 ± 7.29% to 57.13 ± 3.86% in formulations F4 and FL1, respectively. On the basis of the three-dimensional response surface plot, it was shown that production yield was significantly decreased when GEL concentration increased. However, LUL presence in the formulations FL1–FL4 had a significant impact on the production yield increase (Figure 5a).

The particle size analysis showed that the mean diameter of the microparticles was in the range from 1.58 ± 0.13 μm to 2.25 ± 0.26 μm in the placebo formulation and from 1.67 ± 0.17 μm to 2.51 ± 0.60 μm in the LUL-loaded formulations (Table 2). On the basis of the three-dimensional response surface plot, it was observed that particle size was significantly decreased when GEL concentration increased (Figure 5b). The obtained data suggest that the presence of both GEL and LUL in the microparticles increased the particle diameter. This fact might be related to the viscosity of spray-dried solutions—when GEL concentration was increased, ALG/GEL solution viscosity was also increased, and as a consequence, the mean diameter of developed particles was also greater. Higher viscosity of feed solution creates larger droplets as well as leads to formation of larger particles during the spray-drying process. The polydyspersity index (PDI) values of the designed ALG/GEL microparticles was in the range from 0.26 ± 0.12 to 0.40 ± 0.11. It was shown that PDI in all obtained formulations was below 0.7, indicating that the microparticles possessed a narrow size distribution [27]. The similar effect was shown in the work of Tu et al. [28], where GEL/ALG microspheres size was increased with the increase in GEL concentration.

Drug content determination is a crucial parameter affecting dosing of formulations. It was observed that GEL addition did not affect the drug content, but it had a significant effect (*p* < 0.05) on the increase of EE values (from 90.63 ± 3.19% in FL1 to 108.05 ± 7.82% in F4L, Table 2). Similar results were reported by Tu et al., where EE of *Bacillus subtilis* in ALG/GEL beads was significantly increased with increased GEL content in the formulations [28]. The presence of moisture in microparticles is an important parameter enabling prediction of their flow behavior. In addition, the moisture presence in the formulation also affects their stability [29]. It was shown that the spray-drying process enabled the preparation of microparticles, with moisture values in the range from 7.43 ± 2.88% in formulation FL2 to 9.44 ± 2.07% in F4 (Table 2).

### 3.3. Swelling and Mucoadhesive Ability

Ability to swell after contact with moisture is a valid parameter which affects the mucoadhesion process. Mucoadhesion is a complex phenomenon that arises as a result of physical and chemical processes, e.g., covalent bonds, van der Waals forces, hydrogen and hydrophobic interactions, or electrostatic forces. After swelling, polymer chains penetrate the mucosa and form mucoadhesive bonds, enabling intimate drug contact with the mucosa and increasing its concentration at the application site [30,31].

**Figure 6:**
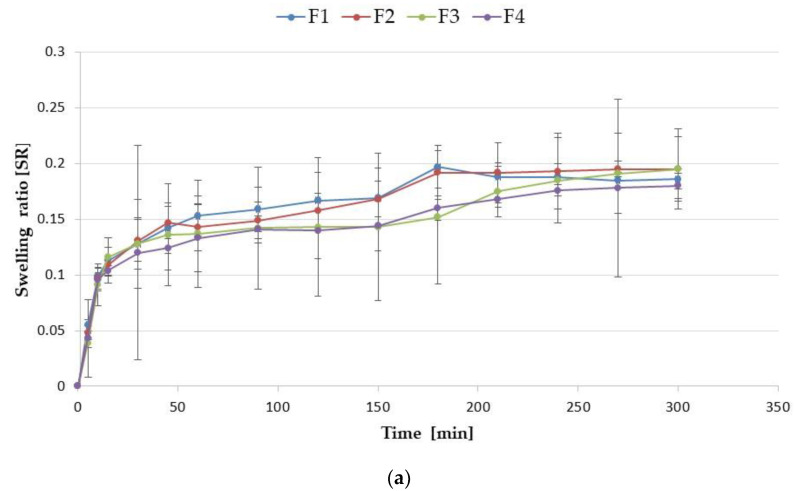

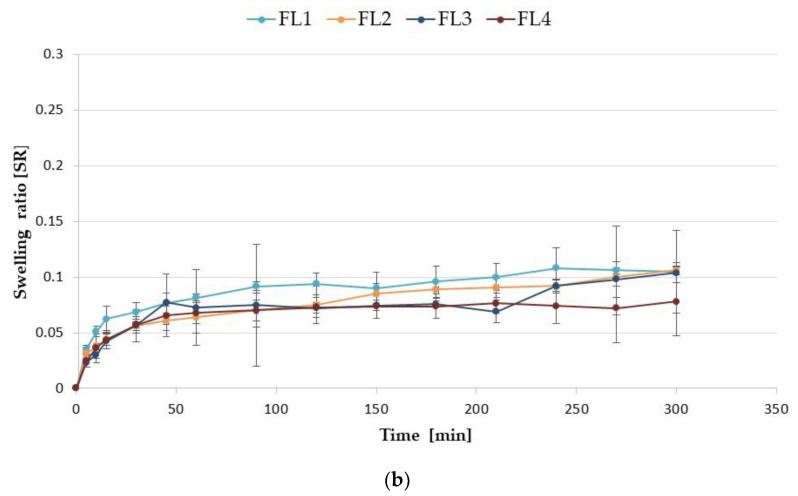
Swelling ratio of (**a**) placebo (F1–F4) and (**b**) LUL-loaded (FL1–FL4) microparticles in SVF at pH 4.2 (mean ± SD, *n* = 3).

To imitate vaginal conditions, the swelling test was performed using SVF (pH 4.2). The swelling ratio (SR) graphs (Figure 6) express that all formulations were characterized by swelling ability due to the high strong hydrophilic nature of ALG and GEL resulting from the presence of large amounts of hydroxyl and carboxylic groups in the polymers chain. Interestingly, it was shown that the GEL addition reduced the microparticle swelling. Formulation F1, composed of only ALG, expressed a high swelling ability and expressed a maximum after 180 min (0.197 ± 0.019), which gradually decreased up to 300 min and reached 0.186 ± 0.009 (Figure 6a) due to polymer dissolution in the medium. However, formulation F4, with high GEL concentration, possessed lower swelling properties, which was presented on the graph as a sharp peak after 15 min, with value 0.104 ± 0.004 and reached 0.180 ± 0.011 after 300 min. It is known that in the case of GEL-based formulations, the pH value of the environment affects the swelling properties. Oh et al. observed that hydrogel composed of synthesized copolymer of acrylic acid and methacrylated gelatin swelled significantly higher under neutral and basic swelling conditions. ALG is an anionic polymer in which carboxylic acid groups at pH values above the pKa (3.21) are ionized, which leads to electrostatic repulsion between the charged groups and to the higher swelling ability [32].

LUL-loaded formulations were characterized by lower SR compared with the placebo, and they reached the values from 0.0232 ± 0.004 (FL3) after 5 min to (FL1) 0.108 ± 0.018 after 240 min (Figure 6b).

**Figure 7:**
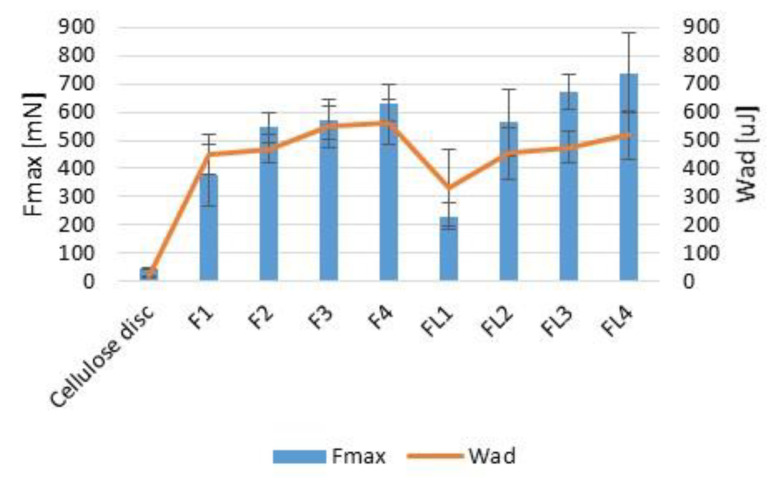
Mucoadhesiveness evaluated using vaginal mucosa presented as detachment force (F_max_) and work of adhesion (W_ad_) of placebo (F1–F4) and LUL-loaded (FL1–FL4) microparticles (mean ± SD, *n* = 6).

Microparticle mucoadhesiveness, presented as detachment force F_max_ (N), and the work of adhesion W_ad_ (μJ), was examined using porcine vaginal mucosa as the model adhesive layer. It was shown that ALG and ALG/GEL microparticles possessed high mucoadhesive ability (Figure 7). The obtained data showed that mucoadhesive properties increased with the increase in GEL concentration (F_max_ from 547.00 ± 52.77 mN in F2 to 632.00 ± 65.34 mN in F4 and W_ad_ from 470.126 ± 48.25 μJ in F2 to W_ad_ 565.17 ± 77.83 μJ in F4). ALG is well-known as a mucoadhesive anionic polymer, with a carboxyl group that might create hydrogen bonds with the hydroxyl groups of mucin glycoproteins. GEL possesses a high level of hydrophilic groups (amine and carboxylate groups) that are responsible for polymer backbone flexibility, which are considered to be crucial for mucoadhesive processes. It was observed that placebo formulations possessed F_max_ from 376.70 ± 111.59 mN in F1 to 632.00 ± 65. 34 mN in F4 and W_ad_ from 450.15 ± 73.49 μJ in F1 to W_ad_ 565.17 ± 77.83 μJ in F4. Similar dependence was also noted by Sahasathian et al. who noticed that GEL presence in ALG/chitosan beads with amoxicillin significantly improved adhesive properties [33]. Kotagale et al. also reported that when GEL addition increased in the carbopol 934-sodium ALG/GEL mucoadhesive ondansetron tablets (from 1:1:1 to 1:1:5), higher bioadhesive strength was observed [34].

It was shown that LUL presence in the formulations diminished interactions of polymers with the adhesive layer by limiting the access of ALG carboxyl groups polymers to the mucosa. As the consequence, the mucoadhesive properties of LUL-loaded formulations were reduced (from F_max_—229.67 ± 48.11 N and W_ad_—332.48 ± 137.25 μJ in formulation FL1 to F_max_ 737.67 ± 140.84 N and W_ad_ 518.94 ± 85.52 μJ in formulation FL4).

**Figure 8:**
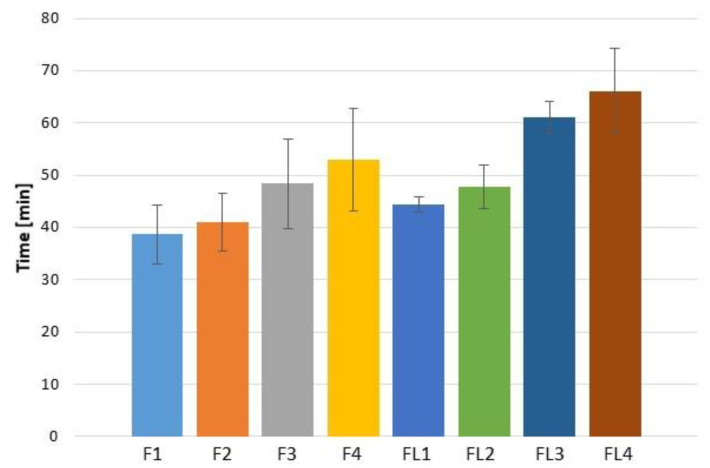
Ex vivo retention time of placebo (F1–F4) and LUL-loaded (FL1–FL4) microparticles (mean ± SD, *n* = 3).

Ex vivo retention time (Figure 8) of prepared ALG/GEL microparticles was examined using porcine vaginal mucosa to simulate the in vivo environment. The ex vivo wash-off time revealed that microparticles attached to the mucosal surface of the vaginal mucosa, which confirmed their mucoadhesive properties. The highest retention time was noticed in formulation FL4 with a value of 66.00 ± 8.18 min. It was observed that formulations of placebo were characterized by lower retention time than LUL-loaded formulations (from 38.67 ± 5.69 min in F1 to 44.33 ± 1.53 min in FL1 and from 53.00 ± 9.85 min in F4 to 66.00 ± 8.19 min in FL4). The possible explanation might be related to LUL poor solubility in the medium, which hindered the water inflow into the matrix, and, as a result, sustained retention time of LUL-loaded microparticles was noted.

### 3.4. In Vitro LUL Release

Although evaluation of drug release profile does not reflect the in vivo conditions, it is a crucial analytical tool applied to investigate and predict formulation behavior. Moreover, an appropriately designed in vitro release profile might deliver details about the dosage form release mechanism and kinetics [35]. Formulations with mucoadhesive properties, and by extension, the connection time with the mucosa, elongate the residence time of the active substance at the area of application, which leads to sustained drug dissolution and improved drug therapeutic activity [6].

**Figure 9:**
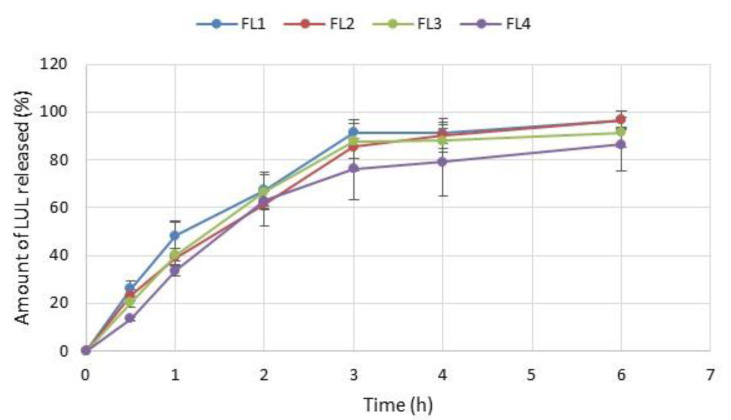
In vitro LUL dissolution studies in SVF pH 4.2 from formulations FL1–FL4 (mean ± SD, n = 3).

In vitro drug release tests from LUL-loaded formulations were performed using SVF (pH 4.2) as an acceptor fluid (Figure 9). Rapid drug burst occurred, so that after 0.5 h, from 13.44 ± 0.63% (in FL4) to 25.98 ± 3.16% (in FL1) of LUL was released. Formulation FL1 was characterized by the fastest drug release (91.31 ± 5.45% of LUL was released after 3 h). A significant (*p* < 0.05) sustained LUL release was noticed in the formulations FL4 (after 6 h 86.50 ± 11.07% of drug was released), which is a result of GEL addition. This fact is related to GEL swelling ability, which affects the drug release profile. Hence, there are an inverse association between swelling capacity and the drug release profile. The drug release process from swellable hydrophilic formulations is controlled by physical and chemical processes, which consist of liquid permeation within the polymer network, the polymer swelling, drug dissolution, and diffusion through the swollen matrix, which might be combined with its degradation. Polymer with lower swelling ability represents a more resistant barrier for the influx of liquid, which reduces drug diffusion [36]. SR values of ALG/GEL microparticles are correlated with the LUL release profile. ALG microparticles possessed high SR values, then they disintegrated. Thus, in formulations containing GEL, which were characterized by lower swelling ability, water inflow into the formulation was reduced, and, as a consequence, LUL dissolution was sustained [37]. This fact is confirmed by the test results obtained by Tran et al., who observed that profile release of ganciclovir form microspheres with higher GEL/drug ratio was more prolonged that from microparticles with lower GEL/drug ratios [26]. A similar result was reported by Farhangi et al., who noted that GEL concentration had a significant effect on the release profile. They observed that microspheres with GEL addition enabled prolonged (even up to 48 h) meloxicam release after intra-articular administration [38].

Results from the release study were applied to the modelling with mathematical equations: zero-order and first-order kinetics, Korsmeyer–Peppas, Higuchi, and Hixson–Crowell models (Table 3), in order to evaluate the LUL release mechanism. The obtained data suggest that drug release was not concentration-dependent, and it was in accordance with the zero-order kinetics. The diffusion exponent (*n*) obtained from the Korsmeyer–Peppas model enables the estimation of the drug release mechanism. Therefore, the obtained *n* values ≤ 0.43 [39] (from 0.31 to 0.41) confirmed diffusion as a mechanism of LUL release from the designed microparticles. These data were also acknowledged by values of R^2^ in the Highuchi equation. Additionally, obtained data from the Hixson–Crowell equation indicated high R^2^ values, which assumes that LUL release was also related to the disintegration of the formulation [23,40]. In conclusion, LUL release from ALG and ALG/GEL microparticles is a complicated process based on erosion and diffusion. Similar data were reported by Shehata et al.—metformin hydrochloride release from ALG/GEL nanoparticles was a coupling of diffusion and erosion mechanisms (anomalous release mechanism) [41].

### 3.5. Thermal Analysis

**Figure 10:**
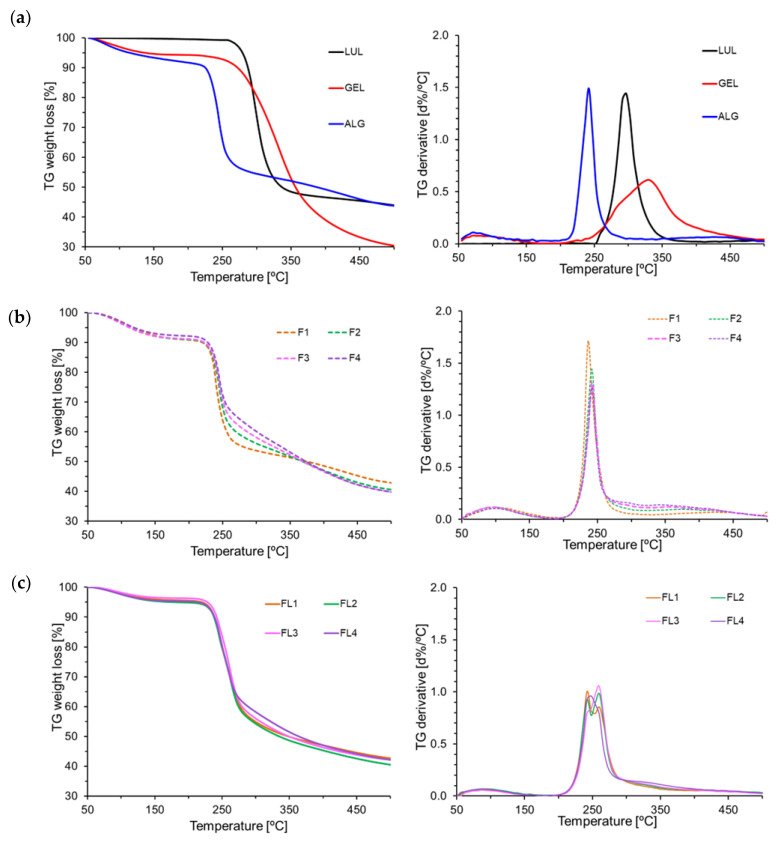
TGA curves of (**a**) ALG, GEL, and LUL, (**b**) placebo formulations (F1–F4), and (**c**) LUL-loaded formulations (FL1–FL4).

Thermogravimetric analysis (TGA) is based on the registration of the sample weight change that occurs when heated at a constant rate. TGA provides information about the thermal stability and behavior of samples [39]. Therefore, thermogravimetric analysis of prepared ALG and ALG/GEL microparticles was conducted (Figure 10). In Figure 10a, the ALG thermogram shows two thermogravimetric steps, with the first being an 8% mass loss at the temperature range from 50 °C to 150 °C, which is associated with dehydration of adsorbed water linked with polymer by hydrogen bonds. The second step of ALG weight loss (42%), which is typically related to the destruction of ALG glycosidic bonds, was observed in the range from 150 °C to 500 °C and corresponded to ALG fragmentation due to the chain breakage [42]. Figure 10a shows two steps of weight loss during the heating of GEL. The first, thermogravimetric step (50–188 °C), showed a 6% weight loss, which might be related to the loss of moisture. The second one (188–500 °C) showed a 67% GEL weight loss, which might be attributed to the thermal breakdown of the protein chain, leading to the elimination of ammonia [43]. Pure LUL was characterized by a one-step decomposition, with a total weight loss of 59%. It was observed that placebo formulations compared with LUL-loaded formulations showed faster weight loss, reaching 90% at 225 °C (Figure 10b). Formulations with the highest GEL content were characterized by a lower weight loss in the range of 250–350 °C, which was confirmed by the thermogram of unprocessed GEL [44]. In addition, it was observed that both ALG and GEL degradation events shifted toward higher temperatures in the microparticles compared with the unprocessed polymers [45]. LUL-loaded formulations were characterized by similar total weight loss compared with the placebo formulations (e.g., from 62% for F4 to 60% for FL4) (Figure 10c).

**Figure 11:**
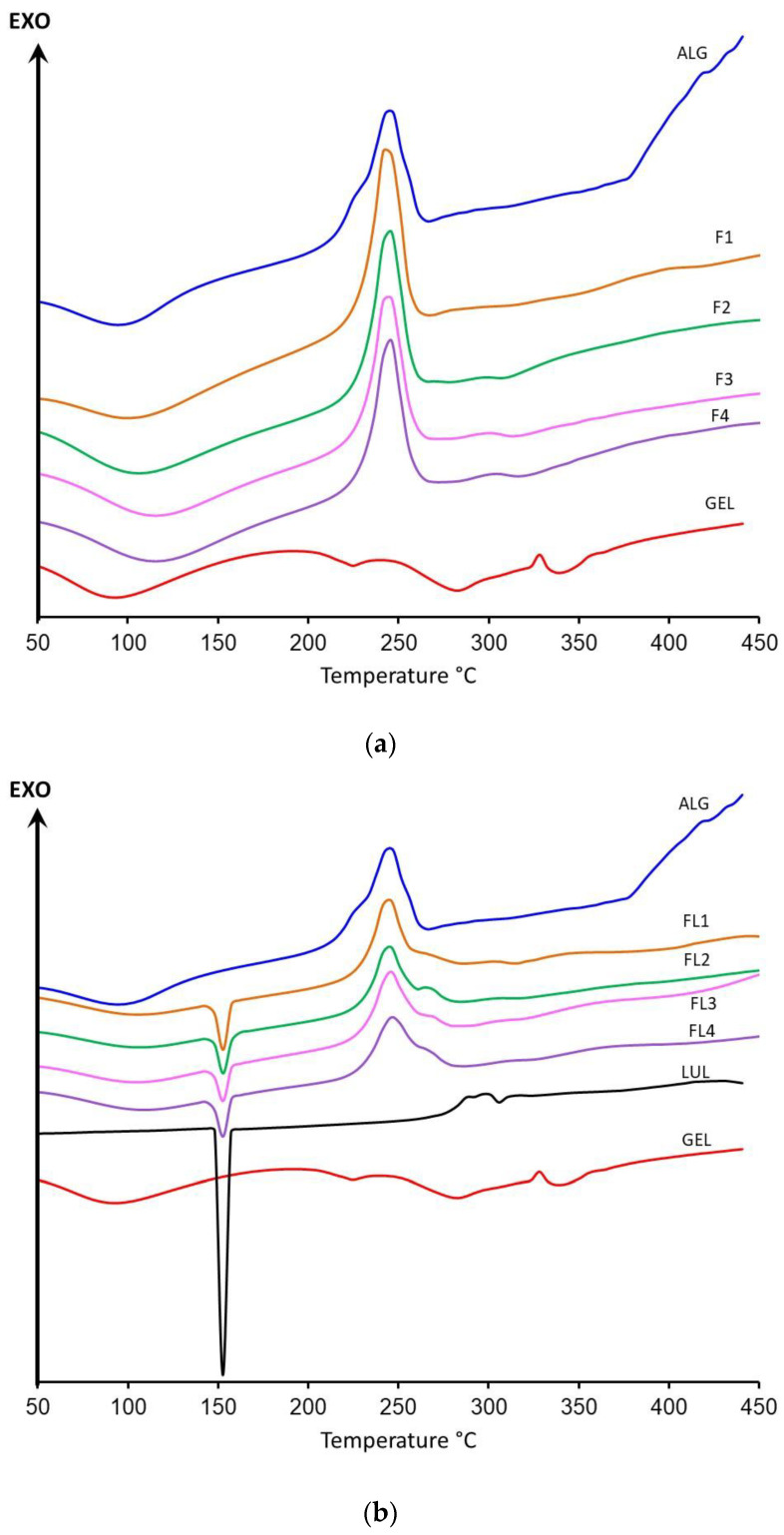
DSC thermograms for (**a**) ALG, GEL, and placebo formulations (F1–F4) and (**b**) ALG, GEL, LUL and LUL-loaded formulations (FL1–FL4).

DSC is a useful analytical tool providing data about the relationship and possible interactions among ingredients of drug dosage forms by analyzing melting points, shifts, occurrence, and leak of peaks and relative peaks area [46]. Figure 11a presents thermograms of ALG, GEL, and placebo formulations F1–F4, and Figure 11b shows LUL-loaded formulations. ALG thermograms exhibit glass transition temperature at 98.7 °C and a wide endothermic peak in the range of 58.2–130.2 °C, correlated with water evaporation. Moreover, a sharp exothermic peak at 248 °C was detected, and it is likely related to the polymer decomposition [47]. Dudek et al. suggested that exothermic peaks of ALG were a result of polymer chain degradation in dehydration, depolymerization, and saccharide ring destruction processes [48]. An exothermic decomposition peak from 377.7 °C to 450 °C indicated sodium carbonate (Na_2_CO_3_) formation [49]. The GEL DSC curve shows the wide endothermic peak in the range of 58.2–152.7 °C, which is related to moisture vaporization. The observed endothermic peak, with a maximum of 224 °C, proves the decomposition of protein likely in the side chain. Additionally, an exothermic peak was noticed, with the maximum at approximately 335 °C, which can be attributed to main chain disintegration [50,51]. The melting point of pure LUL was expressed as a sharp endothermic peak at 152.7 °C, which is comparable to data presented by Kumar et al. [52]. The obtained results suggest that the LUL crystalline state was preserved. The one-step decomposition of LUL in the ambient atmosphere was detectable in the range of 250–350 °C, with the maximum thermal decomposition at 297 °C. Degradation of LUL occurs with the production of low-molecular-weight gaseous products. The DSC curves of placebo formulations exhibited a shift of the maximum of the broad endothermic peak from 101.5 °C to 120.7 °C in formulations F1 and F4, respectively. Peaks of ALG decomposition at 377.7 °C were not observed in microparticles placebo and LUL-loaded formulations, which indicated thermal stability. In DSC thermograms of LUL-loaded formulations, the ALG degradation peak was broadened and was accompanied by a small signal at approximately 270 °C, which might suggest the creation of a complex between LUL and ALG carboxyl groups [52,53].

### 3.6. FTIR–ATR Analysis

**Figure 12:**
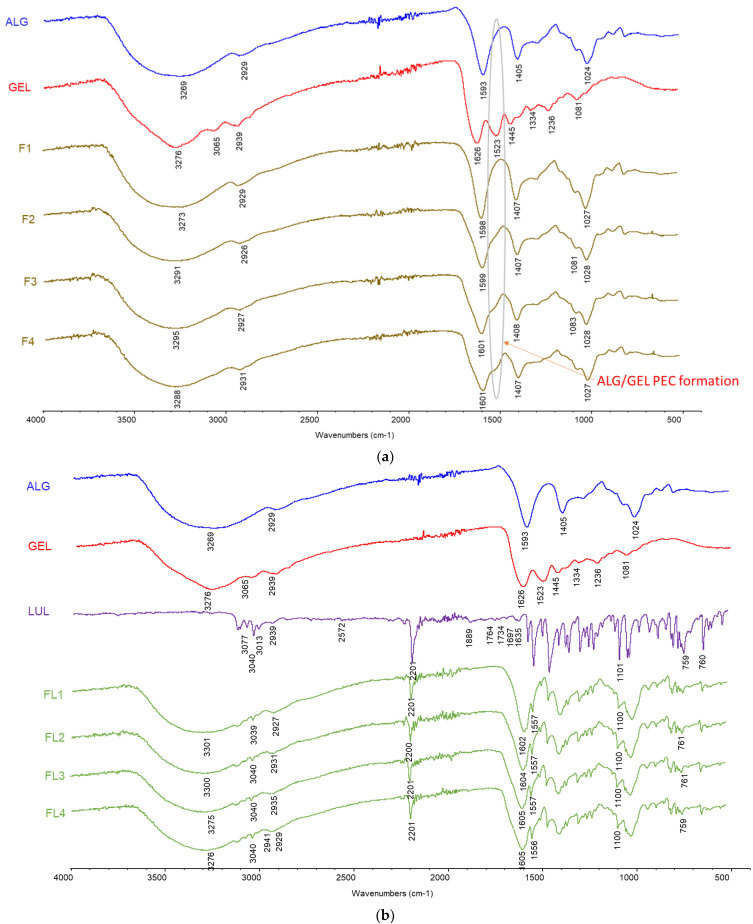
FTIR-AR spectra of (**a**) ALG, GEL, and placebo formulations (F1–F4) and (**b**) ALG, GEL, LUL, and LUL-loaded formulations (FL1–FL4).

Fourier transform infrared spectroscopy–attenuated total reflectance (FTIR–ATR) provides information regarding the appearance or absence of functional groups, shifts in the frequency of bands, and changes in its intensities, which enables the detection of possible interactions between drug and polymer [54]. FTIR–ATR spectra of ALG, GEL, LUL, placebo, and LUL-loaded formulations are shown in Figure 12. In the ALG spectrum, a wide band of stretching vibrations of O-H bonds was observed in the range of 3600–3000 cm^−1^, with a maximum at 3269 cm^−1^, and a band of stretching vibrations of C-H bonds at 2929 cm^−1^. A band of asymmetric vibrations originating from carboxyl salt ions at 1593 cm^−1^, a band of symmetrical vibrations originating from carboxylic salt ions at 1405 cm^−1^, and a band of stretching vibrations of C-O bonds of the pyranosyl ring at 1024 cm^−1^ were also observed. The GEL spectrum presented a wide band of vibrations from the hydrogen bonding of water, with the amide band A at 3276 cm^−1^ and the amide band B at 3065 cm^−1^. The band of stretching vibrations of the C-H bonds at 2939 cm^−1^, the amide band I at 1630 cm^−1^, amide band II at 1523 cm^−1^, a band of symmetrical vibrations of the C-H bond at 1460 cm^−1^, a band of asymmetric vibrations of the C-H bond at 1334 cm^−1^, and amide band III at 1236 cm^−1^ were also detected.

ALG carboxyl groups interact with GEL amino groups and form a complex containing amide. In ALG/GEL placebo microparticles (formulations F2–F4), a peak for amide in the region of 1500–1400 cm^−1^ was observed, which confirmed the reaction among constituent polymers. The intensity of the amide peak was increased with a higher amount of GEL in the formulations. However, the intensities of bands at 1593 cm^−1^ and 1411 cm^−1^ of unprocessed ALG were decreased with the increase in GEL content (Figure 12a). Recorded changes imply the creation of intermolecular interactions between ALG and GEL chains, leading to the formation of a polyelectrolyte complex [54].

In the LUL spectrum, bands of stretching vibrations of aromatic C-H bonds at 3125, 3077, 3040, and 3013 cm^−1^ were observed (Figure 12b). Band of stretching vibrations of C-H bonds at 2939 cm^−1^, a band of stretching vibrations of S-H bonds at 2572 cm^−1^, a band of stretching vibrations of C ≡ N bonds at 2201 cm^−1^, bands of stretching vibrations of C = C bonds at 1889, 1764, 1734, and 1697 cm^−1^, bands of stretching vibrations of C = N bonds at 1635 cm^−1^, band of stretching vibrations of C = C bonds at 1557 cm^−1^, and the bands of stretching vibrations of C-Cl bonds at 1101 cm^−1^ and 760 cm^−1^ were detected. FT-IR spectra of LUL-loaded microparticles (formulations FL1-FL4) exhibited characteristic peaks corresponding to the incorporated drug, especially the characteristic band of C ≡ N bond vibrations at 2201 cm^−1^ (Figure 12b). In the case of LUL-loaded formulations, no band characteristic of GEL was observed, which might be related to the overlap of the ALG and LUL peaks [55,56].

### 3.7. Antifungal Activity

**Figure 13:**
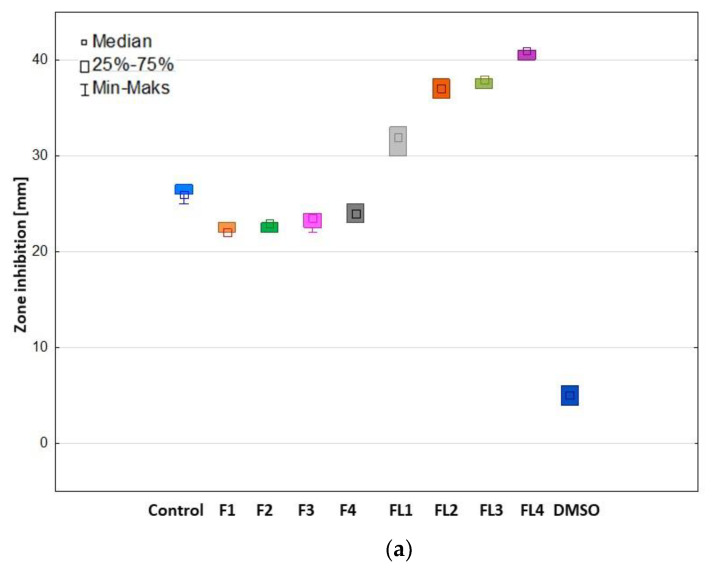

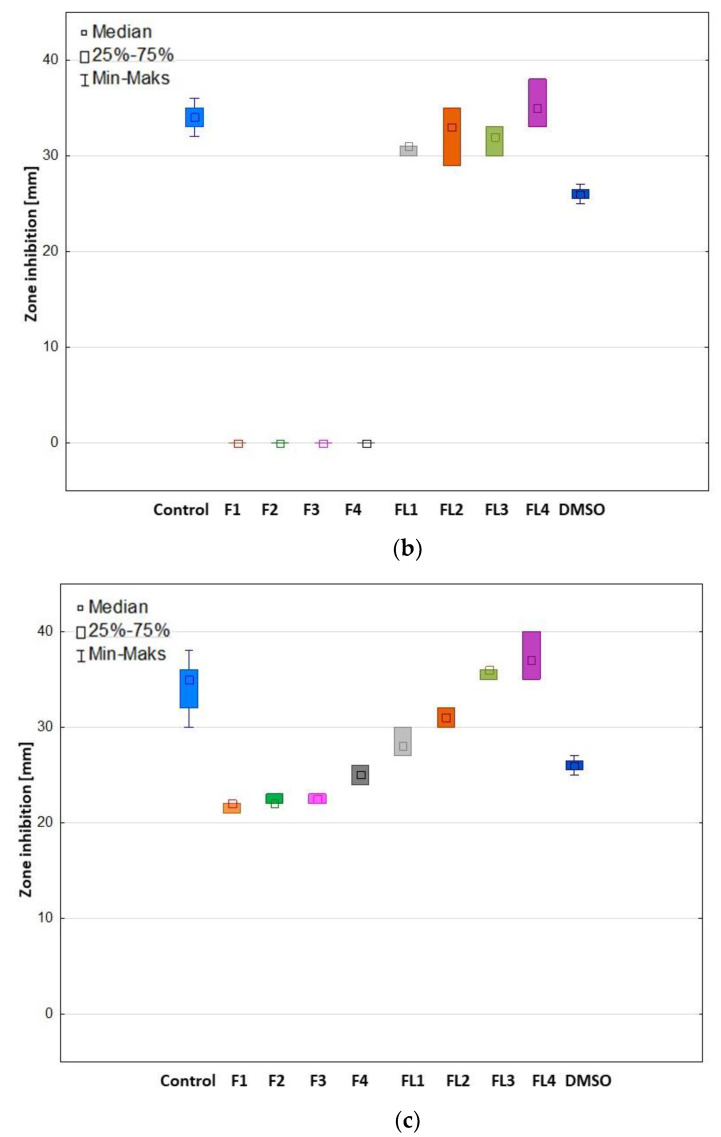
Antifungal action of microparticle placebo formulations (formulations F1–F4) and LUL-loaded microparticles formulations (formulations FL1–FL4); LUL/DMSO (Control) against (**a**) *C. albicans*, (**b**) *C. krusei*, and (**c**) *C. parapsilosis* (*n* = 3).

The developed ALG/GEL microparticles placebo and LUL-loaded microparticles were examined by primary method standardized by Clinical and Laboratory Standards Institute (CLSI)–agar diffusion test for antifungal activity using: *C. albicans*, *C. parapsilosis*, and *C. krusei* strains (Figure 13 and Figure S1) [57]. It was shown that placebo formulations (F1–F4) were characterized by antifungal activity against *C. albicans* (from 22.33 ± 0.58 mm in formulation F1 to 24.17 ± 0.76 mm in F4) and *C. parapsilosis* (from 21.83 ± 0.76 mm in formulation F1 to 25.17 ± 1.04 mm in F4), which indicate that GEL presence in the microparticles improved the antifungal activity in a concentration-dependent manner. The obtained data have shown that all LUL-loaded formulations were characterized by antifungal activity against tested strains, with inhibition zones from 28.33 ± 1.53 mm (FL1) in *C. parapsilosis* to 40.73 ± 0.64 mm (FL4) in *C. albicans* (Figure 13). In the case of LUL-loaded formulations, the highest values of zone inhibition (See Supplementary Materials) were noticed in the *C. albicans* strains (from 31.61 ± 1.53 mm in formulation FL1 to 40.73 ± 0.64 mm in formulation FL4). This fact confirms that with increasing GEL concentration in the formulation, the antifungal activity of microparticles was also increased.

There are many reports indicating ALG antifungal activity per se, which is likely related with the ALG negative charge, providing interaction with the cell membrane and the leakage of the intracellular contents flow outside of the fungal cells. Additionally, ALG disrupts the influx of nutrients through formation of the viscous layer around the fungal cell. ALG might also hinder microbe nutrition by chelating metal, which leads to inhibition of metal-dependent protein production [58,59]. Similar to ALG, GEL also possesses antimicrobial activity related to the formation of a viscous layer on the pathogens cell. This fact was observed by Amrosio et al. in GEL methylene blue loaded nanoparticles—increasing GEL concentration in nanoparticles resulted in higher viscosity of the medium and provided the improvement in the effectiveness of photodynamic chemotherapy against *C. albicans* [60]. Kim et al., who prepared three-dimensional (3D) scaffold type biocomposites of GEL nanoparticles with silver (Ag), also observed positive GEL effect on the antifungal activity. Moreover, there are reports that the 3D scaffold biocomposites of GEL/silver nanoparticles, due to the low oxygen permeability, reduced the hyphae growth of *Aspergillus parasiticus* [61]. Klotz et al. suggested that GEL antifungal activity was based on the inhibition of the adherence by blocking adhesin receptors on the fungus surface. They produced GEL fragments obtained by digestion of the reduced protein with trypsin or cyanogen bromide. Klotz et al. observed that GEL antifungal activity was strictly dependent on the composition of the GEL (GEL with 47 amino acids reduced fungal adherence to type I collagen by 100%). This fact indicates that GEL might possess a crucial role in adhesion inhibition of the fungus cell to host proteins [62].

## 4. Conclusions

ALG/GEL polyelectrolyte complex mixture might be successfully used to develop microparticles by a one-step spray-drying technique. The examination of the impact of GEL addition on the microparticles characteristics showed that GEL presence affected the particles size and improved encapsulation efficiency. Additionally, it was observed that GEL addition prolonged the LUL release, which is a complex process based on drug diffusion and microparticles erosion. GEL presence in microparticles significantly improved the mucoadhesive properties but decreased the swelling capacity. Moreover, the present study expressed that all prepared microparticles hindered the growth of tested *Candida* spp. strains, and GEL presence in the microparticles formulation improved the antifungal activity. The obtained data constitute a valid stage in the design of multicompartment drug carriers for vaginal delivery of antifungals.

## Figures and Tables

**Table 1:** Constitution of ALG/GEL microparticles.

Formulation	ALG Concentration (*w*/*v* %)	GEL Concentration (*w*/*v* %)	LUL Concentration (*w*/*v* %)
F1	1	–	–
F2	1	0.125	–
F3	1	0.250	–
F4	1	0.375	–
FL1	1	–	1
FL2	1	0.125	1
FL3	1	0.250	1
FL4	1	0.375	1

**Table 2:** Quality determination of ALG and ALG/GEL microparticle placebos (F1–F4) and ALG and ALG/GEL LUL-loaded microparticles formulations (FL1–FL4) (mean ± SD, *n* = 3).

Formulation	Production Yield (%)	Particle Size (µm)	Polydyspersity Index (PDI)	Percent Loading (%)	Encapsulation Efficiency (%)	Moisture Presence (%)
F1	55.56 ± 6.79	1.58 ± 0.13	0.27 ± 0.13	–	–	8.87 ± 2.71
F2	54.47 ± 1.97	1.64 ± 0.49	0.40 ± 0.11	–	–	7.68 ± 3.01
F3	51.28 ± 7.63	2.22 ± 1.24	0.28 ± 0.10	–	–	8.22 ± 2.28
F4	50.60 ± 7.29	2.25 ± 0.26	0.32 ± 0.25	–	–	9.44 ± 2.07
FL1	57.13 ± 3.86	1.67 ± 0.17	0.26 ± 0.12	45.32 ± 1.60	90.63 ± 3.19	7.64 ± 2.51
FL2	55.37 ± 4.09	2.15 ± 0.36	0.35 ± 0.15	44.52 ± 0.47	94.62 ± 0.99	7.43 ± 2.88
FL3	55.44 ± 4.19	2.40 ± 0.28	0.38 ± 0.15	44.17 ± 0.63	99.39 ± 1.41	7.54 ± 2.33
FL4	55.96 ± 7.09	2.51 ± 0.60	0.32 ± 0.13	45.50 ± 3.29	108.05 ± 7.82	6.17 ± 1.86

**Table 3:** Models of LUL release from ALG and ALG/GEL microparticles.

Formu- lation	Zero-Order Kinetics		First-Order Kinetics		Highuchi Model		Hixson– Crowell Model		Korsmeyer–Peppas Model		
	R^2^	K	R^2^	K	R^2^	K	R^2^	K	R^2^	K	*n*
FL1	0.75	12.58	0.68	0.25	0.87	43.00	0.80	4.19	0.82	0.43	0.38
FL2	0.68	12.68	0.62	0.23	0.82	43.83	0.83	4.08	0.77	0.43	0.35
FL3	0.83	12.43	0.72	0.21	0.93	41.74	0.94	4.24	0.85	0.41	0.31
FL4	0.74	12.04	0.60	0.27	0.89	41.00	0.89	4.25	0.75	0.44	0.41

R^2^: correlation coefficient, K: release constant, and *n*: the release exponent.

## Data Availability

Data are contained within the article; raw data are available upon request.

## Supplementary Materials

The following supporting information can be downloaded at: https://www.mdpi.com/article/10.3390/ma16010403/s1, Figure S1: Representative images of zone inhibition in (**a**) *Candida albicans*, (**b**) *Candida krusei*, and (**c**) *Candida parapsilosis* strains.
