# Supplementary material for: The Spray-Dried Alginate/Gelatin Microparticles with Luliconazole as Mucoadhesive Drug Delivery System
Source: Materials (Basel). 2023 Jan 1;16(1):403. doi: 10.3390/ma16010403 (PMC9822401; doi:10.3390/ma16010403)
Supplement: Supplementary file 1 [file materials-16-00403-s001.zip › materials-2110769-supplementary.pdf]

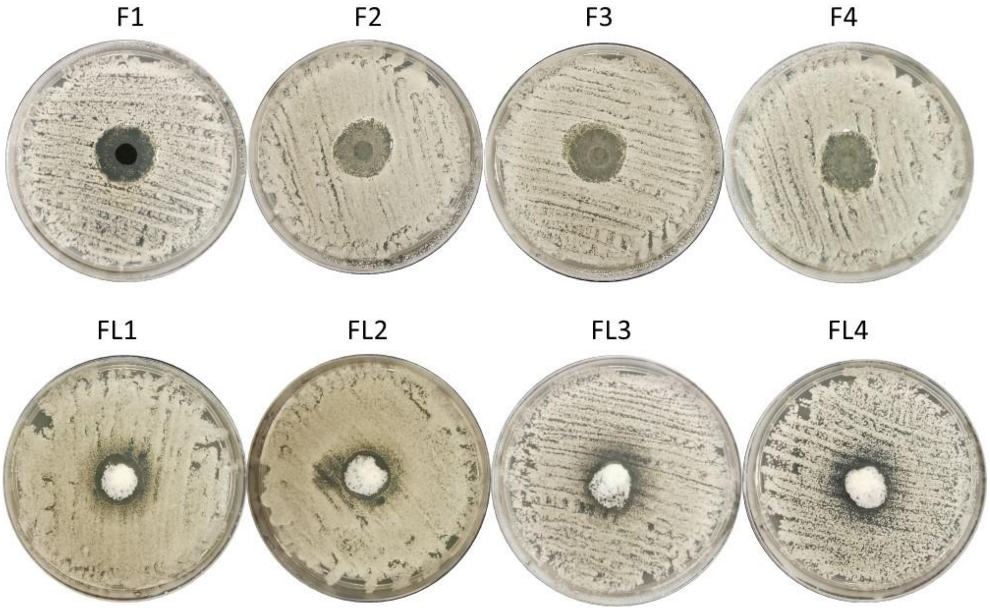

(a)

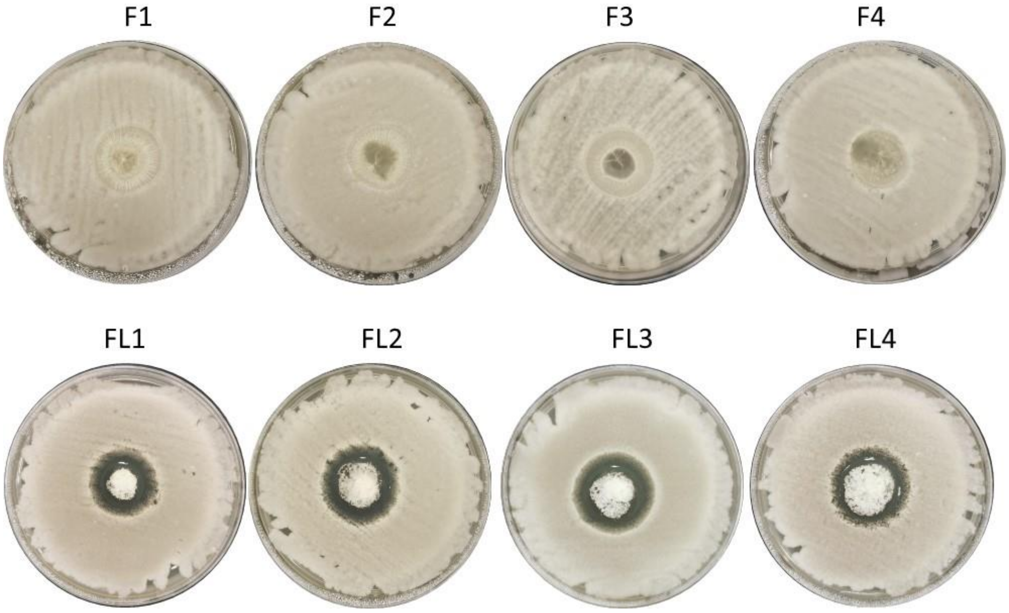

(b)

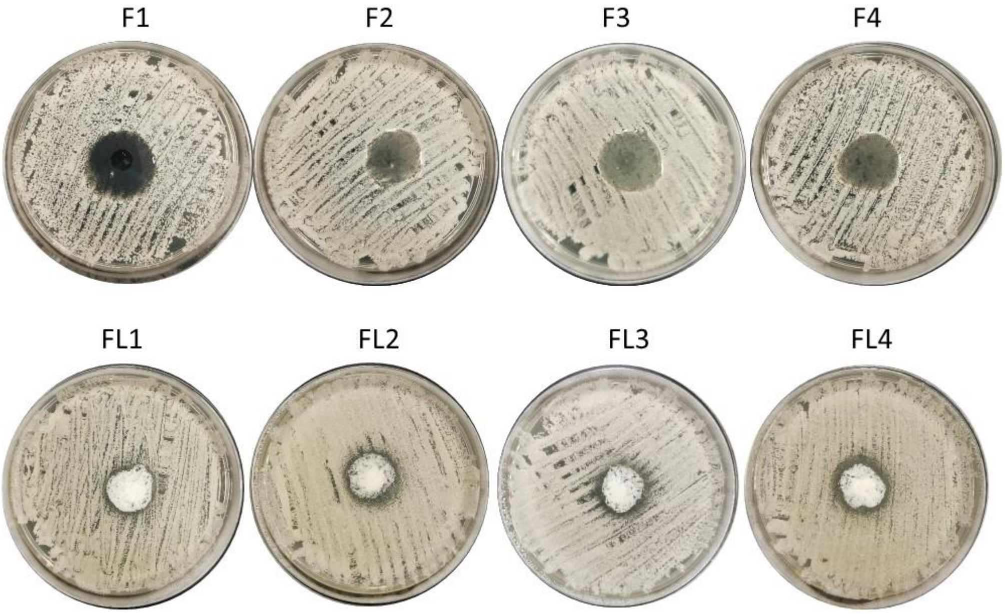

(c)

Figure S1: Representative images of zone inhibition in (a) *Candida albicans*, (b) *Candida krusei* and (c) *Candida parapsilosis* strains.
